# Impact of Family History and Germline Genetic Risk Single Nucleotide Polymorphisms on Long-Term Outcomes of Favorable-Risk Prostate Cancer

**DOI:** 10.1097/JU.0000000000003927

**Published:** 2024-04-10

**Authors:** Florian Rumpf, Anna Plym, Jane B. Vaselkiv, Kathryn L. Penney, Mark A. Preston, Adam S. Kibel, Lorelei A. Mucci, Keyan Salari

**Affiliations:** 1 Department of Urology, Massachusetts General Hospital, Boston, Massachusetts; 2 Department of Anesthesiology, Intensive Care, Emergency, and Pain Medicine, University Hospital Wuerzburg, Wuerzburg, Germany; 3 Department of Epidemiology, Harvard T. H. Chan School of Public Health, Boston, Massachusetts; 4 Division of Urology, Department of Surgery, Brigham and Women’s Hospital, Boston, Massachusetts; 5 Department of Medical Epidemiology and Biostatistics, Karolinska Institutet, Stockholm, Sweden; 6 Channing Division of Network Medicine, Department of Medicine, Brigham and Women's Hospital and Harvard Medical School, Boston, Massachusetts; 7 Center for Genomic Medicine, Massachusetts General Hospital, Boston, Massachusetts; 8 Broad Institute of The Massachusetts Institute of Technology and Harvard, Cambridge, Massachusetts

**Keywords:** prostate cancer, active surveillance, family history, hereditary cancer syndrome, germline risk variants

## Abstract

**Purpose:**

Family history and germline genetic risk single nucleotide polymorphisms (SNPs) have been separately shown to stratify lifetime risk of prostate cancer. Here, we evaluate the combined prognostic value of family history of prostate and other related cancers and germline risk SNPs among patients with favorable-risk prostate cancer.

**Materials and Methods:**

A total of 1367 participants from the prospective Health Professionals Follow-up Study diagnosed with low- or favorable intermediate-risk prostate cancer from 1986 to 2017 underwent genome-wide SNP genotyping. Multivariable Cox regression was used to estimate the association between family history, specific germline risk variants, and a 269 SNP polygenic risk score with prostate cancer‒specific death.

**Results:**

Family history of prostate, breast, and/or pancreatic cancer was observed in 489 (36%) participants. With median follow-up from diagnosis of 14.9 years, participants with favorable-risk prostate cancer with a positive family history had a significantly higher risk of prostate cancer‒specific death (HR 1.95, 95% CI 1.15-3.32, *P* = .014) compared to those without any family history. The rs2735839 (19q13) risk allele was associated with prostate cancer‒specific death (HR 1.81 per risk allele, 95% CI 1.04-3.17, *P* = .037), whereas the polygenic risk score was not. Combined family history and rs2735839 risk allele were each associated with an additive risk of prostate cancer‒specific death (HR 1.78 per risk factor, 95% CI 1.25-2.53, *P* = .001).

**Conclusions:**

Family history of prostate, breast, or pancreatic cancer and/or a 19q13 germline risk allele are associated with an elevated risk of prostate cancer‒specific death among favorable-risk patients. These findings have implications for how family history and germline genetic risk SNPs should be factored into clinical decision-making around favorable-risk prostate cancer.

## Graphical abstract

**Figure:**
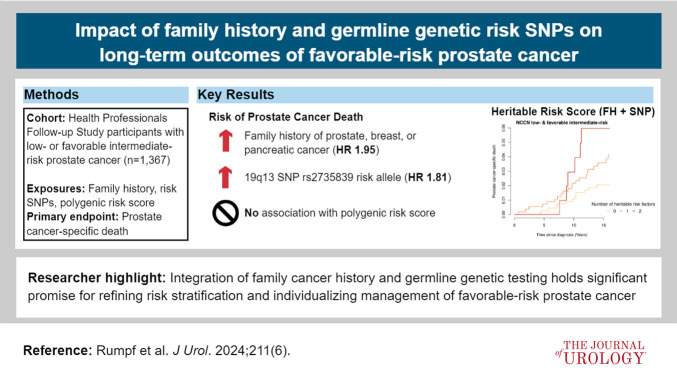

Prostate cancer is the leading cause of cancer-related death among men in 54 countries, and in the US leads to 34,500 deaths annually.^1^ Despite this high disease burden, three-quarters of patients diagnosed are clinically localized,^1^ many of whom have favorable-risk tumors that may not warrant treatment. To mitigate the risks of overtreatment, the AUA and National Comprehensive Cancer Network (NCCN) guidelines recommend active surveillance (AS) as the preferred management strategy for low-risk prostate cancer and an option to consider for favorable intermediate-risk disease.^2,3^ However, there is considerable clinical heterogeneity among patients with favorable-risk prostate cancer, and up to 50% of patients initially on AS eventually require treatment over long-term follow-up.^4,5^ It has long been established that a family history of prostate cancer increases the risk of prostate cancer diagnosis and mortality 2- to 4-fold.^6,7^ There is growing evidence that men with a family history of other cancer types, specifically breast, ovarian, and pancreatic cancer, also may have an increased risk of lethal prostate cancer.^8-13^ While both genetic and environmental risk factors are shared among family members, twin studies attribute > 50% of the variation of prostate cancer risk to genetic factors.^14,15^ Despite these data, current risk stratification strategies and selection criteria for AS rely primarily on clinical and pathologic factors and do not consider family history or germline genetic risk.

The established germline genetic risk factors for prostate cancer range from rare pathogenic mutations in DNA repair genes (eg, *BRCA1/2*) to common single nucleotide polymorphisms (SNPs) associated with prostate cancer risk.^16,17^ To date, > 260 prostate cancer‒associated SNPs have been validated through large-scale genome-wide association studies (GWAS) and used to generate polygenic risk scores (PRSs) in multiethnic populations that stratify risk of developing prostate cancer.^17-19^ While not prognostic, a high PRS increases lifetime risk of high-grade and lethal prostate cancer.^20,21^ Studies investigating genetic association with aggressive prostate cancer have identified only a small number of common variants (eg, loci in the 8q24 region^22,23^ and 19q13 region^24-28^) consistently associated with aggressive disease and/or prostate cancer‒specific mortality. Notably the *KLK3* gene, which encodes for PSA, is located within the 19q13 region.^29^

Few studies have evaluated germline genetic risk SNPs as potential predictors of aggressive disease among patients eligible for or on AS,^26,30,31^ and even fewer studies have examined family history as a predictor of aggressive disease in AS or AS-eligible patients.^32^ Previous studies primarily focused on family history of prostate cancer alone and did not expand the definition of family history to include genetically related cancers such as breast or pancreatic cancer. To our knowledge, no prior study has examined the combined prognostic value of family history and germline genetic risk SNPs among patients with favorable-risk prostate cancer eligible for AS with prostate cancer death as an end point.

Here, we evaluated the combined effect of a positive expanded family history (including not only prostate cancer, but other genetically related cancers) and germline risk SNPs on long-term outcomes of favorable-risk patients. We hypothesized that among patients with favorable-risk prostate cancer eligible for AS, those at elevated risk based on family history and/or germline genetic risk SNPs carry a greater risk of prostate cancer‒specific death.

## MATERIALS AND METHODS

### Study Population

The Health Professionals Follow-up Study (HPFS) is an ongoing prospective cohort study of US male 51,529 health professionals aged 40 to 75 years when enrolled in 1986. Detailed methods have been published elsewhere.^33-37^ Briefly, participants completed detailed questionnaires on their medical history and lifestyle at enrollment and every 2 years thereafter. Participants who indicated they have been diagnosed with prostate cancer throughout follow-up were sent additional prostate cancer‒specific questionnaires biennially starting in 2010. After more than 30 years of follow-up, each cycle has maintained a response rate of > 90% of the remaining active participants.^38^ The deaths of participants were tracked using information from next of kin, the US Postal Service, and the National Death Index, with a previously reported sensitivity of > 98%.^39^

**Figure 1.**
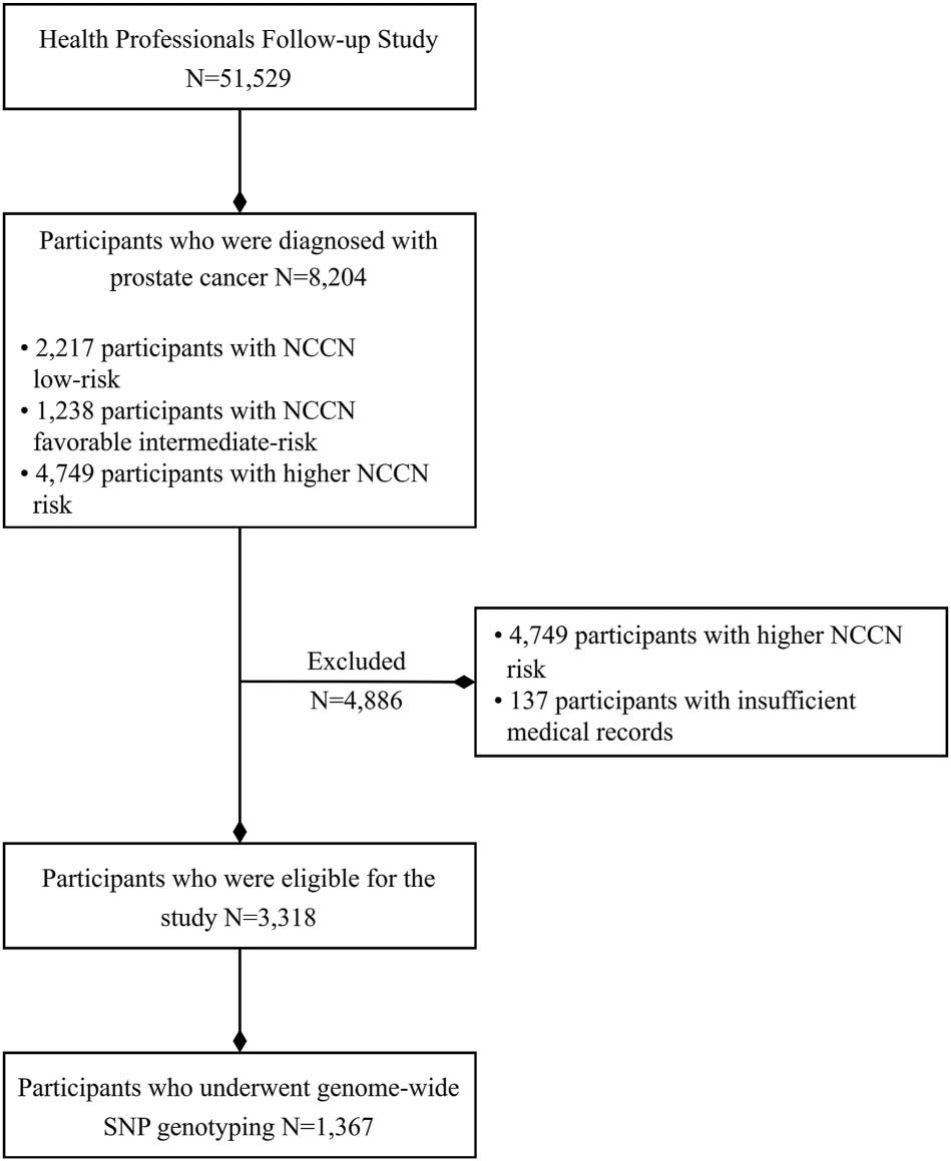
Flow diagram of study population. Starting from 51,529 male health professionals originally enrolled in 1986, 8204 participants have been diagnosed with prostate cancer through 2017. A total of 4749 participants were excluded for higher National Comprehensive Cancer Network (NCCN) risk, and 137 participants had insufficient medical records (age, Gleason grade, clinical stage, PSA, smoking status, family history, survival time, and/or incomplete follow-up). Among 3318 participants deemed eligible for active surveillance, 1367 have been genotyped and were included in all stages of the analysis. SNP indicates single nucleotide polymorphism.

Detailed clinical information related to the cancer diagnosis, such as stage, grade, and pretreatment PSA level, was abstracted from medical records (ie, clinical notes, pathology reports) shortly after the time of diagnosis once a medical records release was obtained. We classified participants diagnosed with prostate cancer according to risk stratification criteria from the NCCN Guidelines for Prostate Cancer to identify patients that would be considered eligible for AS (ie, clinically low-risk and favorable intermediate-risk patients^3^). Low-risk disease was defined as clinical stage T1-T2a, Grade Group 1, and PSA < 10 ng/mL at diagnosis. Favorable intermediate-risk disease was defined as clinical stage T2b-T2c, Grade Group 2, or PSA 10 to 20 ng/mL at diagnosis. The CAPRA (Cancer of the Prostate Risk Assessment) score was evaluated as a second nomogram to minimize the number of covariates.^40^ For both the NCCN and CAPRA score the percentage of biopsy cores positive was omitted because detailed data on diagnostic biopsy parameters were not available for > 85% of participants. Among the 8204 patients diagnosed with prostate cancer in the cohort through January 2017, 2217 participants were classified as low risk and 1238 participants as favorable intermediate risk. We excluded 4749 participants with NCCN unfavorable intermediate-risk, high-risk, or very high-risk disease, and 137 participants with missing information from the medical records (Figure 1). Of the 3318 participants eligible, 1367 had genotyping data available for this study and were included in all stages of the analysis. The study protocol was approved by the Institutional Review Boards at Brigham and Women’s Hospital and Harvard T. H. Chan School of Public Health, and those of participating registries as required.

### Family History

Participants reported first-degree family members diagnosed with cancer and their age at diagnosis on several of the biennial questionnaires. Family history of prostate cancer was part of the 1990, 1992, and 1996 questionnaires, and the supplemental 1994 mothers’ questionnaire. Family history of breast cancer was part of the 1996 questionnaire, and family history of pancreatic cancer was part of the 2008 questionnaire. Family history of ovarian cancer has not been assessed in any questionnaires and thus was not investigated in this analysis. A positive expanded family history was defined as participants with 1 or more first-degree relatives with prostate, breast, and/or pancreatic cancer. The age at diagnosis of first-degree relatives with each cancer type was dichotomized into early and late onset based on conventional age thresholds in the literature^13,41,42^; early onset was defined as age at diagnosis < 60 years for prostate cancer and < 50 years for breast and pancreatic cancer. In cases of multiple family members with cancer, the age of the youngest affected first-degree relative was considered. In 71 cases the age of the youngest family member at cancer diagnosis could not be determined due to missing data. For all measures of family history, the reference was defined as no family history of any of the above cancer types.

### Genotyping

All study participants were invited to provide a blood sample between 1993 and 1995. Between 2004 and 2006, all active study participants who had not previously provided blood samples were invited to provide a buccal cell sample. In this study, 91% of participants were sampled in the initial round and the remainder in the second round. Since 2007, GWAS of 12 different disease outcomes have been conducted using participants from the HPFS. Genotyping was performed on 5 platforms and methods have been previously described.^38^ Germline testing for rare pathogenic mutations (eg, *BRCA1/2*) has not been performed for this cohort. The PRS for prostate cancer risk was computed using a previously validated multiancestry PRS derived from 269 known SNPs, of which 264 were available for analysis.^19^ Two specific prostate cancer risk variants were individually evaluated given their previously reported association with aggressive disease: rs1447295 on 8q24 and rs2735839 on 19q13.

### Statistical Analysis

The primary end point was prostate cancer‒specific death, which was modeled as a competing risk with death due to other causes. Cause-specific hazards for the primary end point were estimated in all models.^43^ Follow-up started from the date of prostate cancer diagnosis to the date of outcome or censoring. Follow-up time was left-truncated for patients diagnosed with prostate cancer before samples for genotyping were collected to minimize immortality bias. In 5 cases the date of sample collection could not be determined and was imputed with the median date for blood collection. Censoring was defined as death from other causes or end of follow-up as of January 2020. Summary statistics were reported as median and interquartile range for continuous variables and proportions for discrete variables. Association between predictors and outcomes was evaluated with multivariable Cox proportional hazards regression models while controlling for age at diagnosis, PSA, Grade Group, and clinical T stage. A model consisting of only these covariates was called the base model. To account for potential overfitting, models were evaluated by 10-fold cross-validated C-index. Furthermore, models with nomograms were compared to models with separate covariates to minimize the number of covariates. When these models were very similar, the individual covariates instead of nomograms were evaluated to enhance clinical applicability. Family history data were included as time-varying covariates to account for the varying time points of family history assessment for each cancer type throughout the study and limit immortality bias. When germline variants were evaluated, the models were also adjusted for the first 3 principal components to account for potential population stratification. The risk of PRS on prostate cancer‒specific death was estimated by modeling PRS as a continuous variable with a restricted cubic spline to account for nonlinear effects. We developed a heritable risk score (range: 0-2) wherein 1 point was given for each of a positive expanded family history and the presence of the 19q13 (rs2735839) risk allele. Risk estimates were reported as cause-specific hazard ratios with 95% CIs. The survival probabilities were computed with the Aalen-Johansen estimator.^43^ Cox models were compared using the LR test. Subgroup analysis was carried out for NCCN low-risk patients with an identical modeling approach. Graphing and statistical analysis was done with R software version 4.1.3 This study is reported following the STROBE (Strengthening the Reporting of Observational Studies in Epidemiology) guideline.

## RESULTS

The study included 1367 men with germline genotype data available who were diagnosed with NCCN low-risk or favorable intermediate-risk prostate cancer during the study period and were included in the analysis (Figure 1). Participants with and without a positive expanded family history had overall similar baseline clinical and demographic characteristics (Table 1). The median age at the time of diagnosis was 69 years (IQR 64-74). At diagnosis, the median PSA was 5.9 ng/mL (IQR 4.5-8.1) and 81% of participants presented with Grade Group 1 prostate cancer. By NCCN risk stratification, 896 (66%) participants had low-risk disease. Only 135 (9.9%) participants elected AS or watchful waiting as the primary management strategy in this largely treated cohort. During the median event-free follow-up from diagnosis of 14.9 years (IQR 11.2-18.8), 55 participants died of prostate cancer.

**Table 1.** Clinical and Demographic Characteristics of Patients With Prostate Cancer in the Health Professionals Follow-Up Study

	Negative family history (N = 878)	Positive family history (N = 489)
Age at diagnosis, median (IQR), y	70 (64, 74)	68 (63, 73)
Race, No. (%)		
White	860 (98)	476 (97)
Asian-American	1 (0.11)	1 (0.20)
Other	10 (1.1)	7 (1.4)
Missing	7 (0.80)	5 (1.0)
Ashkenazi Jewish heritage, No. (%)		
Positive	92 (10)	47 (9.6)
Missing	9 (1.0)	6 (1.2)
BMI at diagnosis, median (IQR), kg/m^2^	25.1 (23.4, 27.3)	25.4 (23.3, 27.9)
Alcohol cumulative average at diagnosis, median (IQR), g/d	8.1 (2.4, 18.0)	7.9 (1.8, 16.4)
Smoking status at diagnosis, No. (%)		
Never	437 (50)	263 (54)
Past, quit >10 y ago	357 (41)	181 (37)
Past, quit ≤10 y ago	62 (7.1)	34 (7.0)
Current	22 (2.5)	11 (2.3)
Smoking pack-years at diagnosis, median (IQR)	0.0 (0.0, 16.0)	0.0 (0.0, 16.0)
5-alpha reductase inhibitor use before diagnosis, No. (%)	34 (3.9)	23 (4.7)
PSA, median (IQR), ng/mL	5.9 (4.5, 8.1)	5.8 (4.5, 8.1)
Grade Group 2, No. (%)	178 (20)	85 (17)
Clinical stage T2, No. (%)	233 (27)	152 (31)
NCCN favorable intermediate-risk, No. (%)	309 (35)	162 (33)
Event-free follow-up, median (IQR), y	14.8 (10.8, 18.6)	15.0 (12.1, 19.2)
Primary treatment, No. (%)		
WW/AS	95 (11)	40 (8.2)
RP	381 (43)	252 (52)
XRT	349 (40)	164 (34)
Other	34 (3.9)	24 (4.9)
Missing	19 (2.2)	9 (1.8)

Abbreviations: AS, active surveillance; NCCN, National Comprehensive Cancer Network; RP, radical prostatectomy; WW, watchful waiting; XRT, radiation therapy.

**Figure 2.**
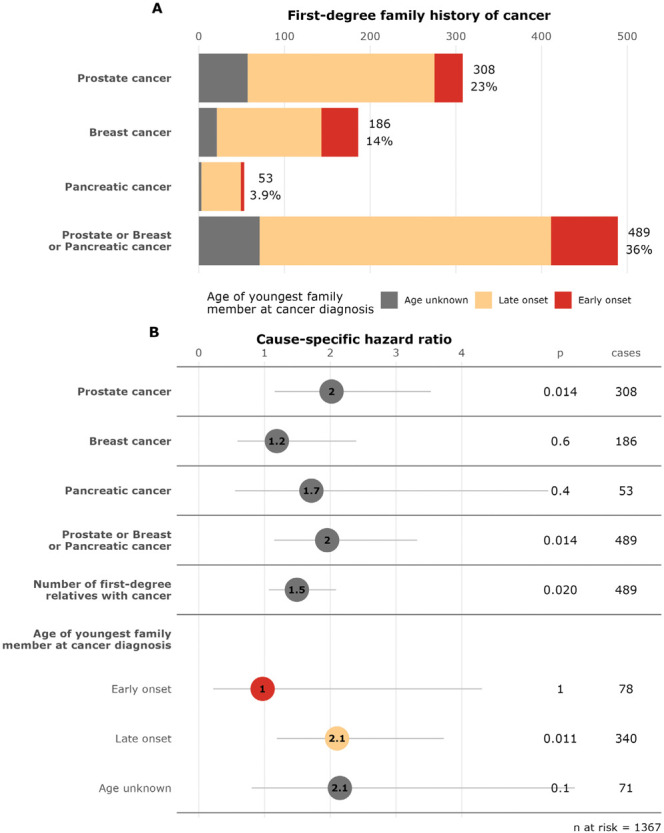
Association of expanded family history and prostate cancer‒specific death. A, Distribution of participants with a family history of cancer in first-degree relatives colored by age of youngest family member at cancer diagnosis. B, Forest plot of associations between family history and prostate cancer‒specific death adjusted for age, PSA, Grade Group, and clinical T stage.

Family history was positive for prostate cancer in 308 (23%) participants, breast cancer in 186 (14%) participants, and pancreatic cancer in 53 (3.9%) participants among first-degree relatives (Figure 2, A). Fifty-eight (4.2%) participants had 2 first-degree relatives with distinct cancers (eg, father with prostate cancer and mother with breast cancer), while no participant had all 3 cancer types in their family history. A family history of prostate cancer alone was positively associated with prostate cancer‒specific death after adjusting for known clinical predictors, whereas a family history of breast or pancreatic cancer each alone was not significantly associated (Figure 2, B). A positive expanded family history, defined as 1 or more first-degree relatives with prostate, breast, and/or pancreatic cancer, was present in 489 (36%) participants. Men with a positive expanded family history carried a significantly higher risk of prostate cancer‒specific death compared to those with no family history, after adjusting for age, PSA, grade, and stage (HR 1.95, 95% CI 1.15-3.32, *P* = .014; Figure 2, B). Notably, the model that included an expanded family history performed significantly better than the base model (likelihood-ratio [LR] test *P* = .042), whereas the model with a family history of prostate cancer alone improved on the base model but to a slightly lesser degree (LR test *P* = .052). The number of first-degree relatives with any of the 3 cancer types was also positively associated with prostate cancer‒specific death (per family member HR 1.49, 95% CI 1.07-2.09, *P* = .020; Figure 2, B). A family history of early-onset prostate (<60 years old), breast (<50 years old), or pancreatic (<50 years old) cancer in first-degree relatives was not associated with a higher risk of prostate cancer‒specific death. Late onset of family history compared with absence of family history was associated with a higher risk of prostate cancer‒specific death but was within the range of risk associated with any positive family history.

**Figure 3.**
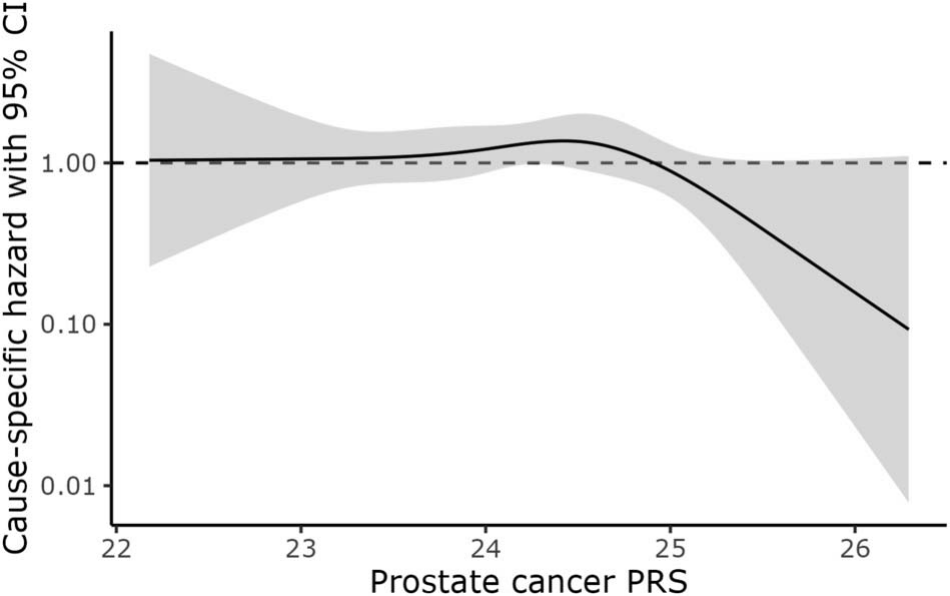
Association between the prostate cancer polygenic risk score (PRS) and prostate cancer‒specific death. The gray ribbon indicates 95% CIs around the cause-specific hazards on a logarithmic scale. The x-axis is limited to PRSs in our data.

We next investigated whether germline genetic risk SNPs for prostate cancer were associated with lethal prostate cancer. We first evaluated a previously validated multiethnic PRS for overall prostate cancer risk derived from 269 SNPs. The PRS was not associated with prostate cancer‒specific death (Figure 3 and Supplementary Table 1, https://www.jurology.com). We then focused on 2 specific risk variants, located on 8q24 (rs1447295) and 19q13 (rs2735839), that have been previously identified and validated as predictors of aggressive disease.^22,23,26,27,31,44^ In the cohort, risk allele frequencies were slightly higher than in the underlying GWAS (Supplementary Table 2, https://www.jurology.com). While the 8q24 risk variant was not associated with prostate cancer‒specific death in our favorable-risk cohort (HR 1.19 per 1 risk allele, 95% CI 0.66-2.14, *P* = .6; Supplementary Table 3, https://www.jurology.com), we found the rs2735839 risk allele on 19q13 was associated with prostate cancer‒specific death (HR 1.81 per 1 risk allele, 95% CI 1.04-3.17, *P* = .037; Supplementary Table 4, https://www.jurology.com).

**Figure 4.**
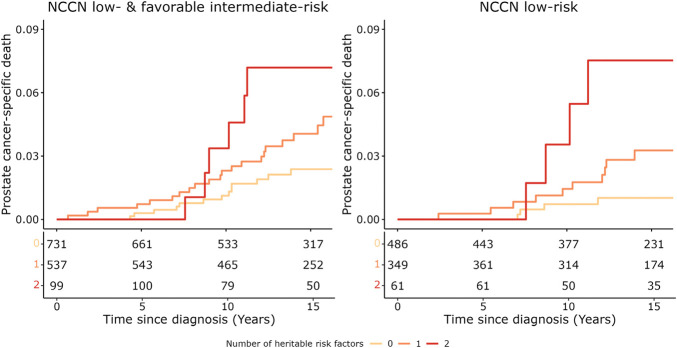
Survival curves from the Aalen-Johansen estimator for prostate cancer‒specific death stratified by number of heritable risk factors among participants with National Comprehensive Cancer Network (NCCN) low- and favorable intermediate-risk prostate cancer (n = 1367; A) and the subgroup of participants with NCCN low-risk prostate cancer (n = 896; B).

Finally, we evaluated the combined effect of both family history and germline genetic risk SNPs. Including both family history and rs2735839 as separate predictors in a multivariable model, we found the association between expanded family history and prostate cancer‒specific death is independent of rs2735839 risk allele dosage (Supplementary Table 5, https://www.jurology.com). We then generated a heritable risk score giving 1 point for each heritable risk factor: a positive expanded family history and rs2735839 risk allele. In multivariable analysis, each point of the heritable risk score was associated with a 78% increased risk of prostate cancer‒specific death (HR 1.78 per point, 95% CI 1.25-2.53, *P* = .001; Table 2, Figure 4, A, and Supplementary Table 6, https://www.jurology.com). Prostate cancer‒specific survival time was shorter for participants with more heritable risk factors (Figure 4, A). This association was even more pronounced for the subgroup of participants with NCCN low-risk disease (Figure 4, B), who we considered meeting stricter criteria for AS. In the NCCN low-risk subgroup, each additional heritable risk factor was associated with a 179% increased risk of prostate cancer‒specific death (HR 2.79 per point, 95% CI 1.57-4.94, *P* < .001; Figure 4, B and Supplementary Table 7, https://www.jurology.com).

**Table 2.** Cause-Specific Hazard Ratios and 95% Confidence Intervals From Multivariable Cox Regression Models of the Heritable Risk Factors and Clinical Factors for Prostate Cancer‒Specific Death in Low-Risk and Favorable Intermediate-Risk Participants of the Health Professionals Follow-Up Study (n = 1367)

Variable	Cause-specific HR	95% CI	*P* value
Age	1.06	1.02-1.11	.007
PSA	1.13	1.06-1.21	< .001
Grade Group 2 vs 1	2.89	1.57-5.33	< .001
Clinical stage T2 vs T1	2.43	1.40-4.21	.002
Risk score: any first-degree relative (0/1) + 19q risk allele (0/1)	1.78	1.25-2.53	.001

The 10-fold cross-validation C-index for the model was 0.73. Sixteen prostate cancer‒specific deaths occurred in patients with a risk score of 0, 32 deaths in patients with a risk score of 1, and 7 deaths in patients with a risk score of 2.

## DISCUSSION

We found that among men in the HPFS cohort diagnosed with favorable-risk prostate cancer, those with a positive expanded family history and/or 19q13 germline risk allele carried an elevated risk of prostate cancer‒specific death. Participants with increasing number of heritable risk factors (from 0-2) had increasingly higher cumulative incidence of prostate cancer‒specific death, particularly among NCCN low-risk patients. The associations are independent of effects of these factors on clinical characteristics of the cohort.

Our study represents one of the largest cohorts, to our knowledge, in which the combined impact of family history and germline genetic risk SNPs on outcomes on AS-eligible prostate cancer has been investigated. Unique to our study is the long-term follow-up allowing investigation of prostate cancer‒specific death as a primary end point and our broader definition of family history inclusive of other malignancies with shared genetic underpinnings with prostate cancer.^9-13^

While previous studies have concluded that family history of prostate cancer is not a risk factor for disease progression among patients eligible for or on AS,^32^ these studies were limited by a narrower definition of family history of prostate cancer alone; we recently showed that when an expanded definition of family history is considered, patients with family history suggestive of a hereditary cancer syndrome have higher rates of biopsy progression on AS.^45^ Our findings of the absence of association between the 269-variant PRS and prostate cancer outcomes are largely consistent with a recent study investigating genetic factors associated with conversion from AS to treatment.^30^ The authors reported some evidence of association with conversion from AS to treatment comparing the highest and lowest deciles of the 269-variant PRS to the middle 2 deciles, whereas all other PRS deciles were not significantly associated with conversion.^30^ Additionally, the primary outcome in this study was conversion from AS to treatment, which introduces several sources of potential bias such that many patients who converted to treatment may not have had truly aggressive or lethal disease. The lack of association we found between the PRS and the long-term outcome of prostate cancer‒specific death in patients with prostate cancer is most consistent with our a priori hypothesis that the current PRS derived from GWAS of incident prostate cancer cases vs controls may stratify risk of developing any prostate cancer but does not distinguish between the risk of indolent vs aggressive disease. However, our study also suggests that certain germline risk SNPs (rs2735839) might be useful for AS risk stratification, and this aligns with findings from previous studies that showed an increased incidence of lethal prostate cancer in at-risk populations (defined by the current PRS).^20,21,30^

The SNP rs2735839 is located near the *KLK3* locus encoding PSA and has been previously reported to influence PSA levels in both healthy individuals and prostate cancer.^29^ Carriers of the major allele (G) have been shown to exhibit raised PSA expression, and thereby contemporary PSA screening could lead to overrepresentation of rs2734839 carriers in prostate cancer patients.^46,47^ This detection bias could result in an inverse association with aggressive status. However, most family history cases in our study date from before PSA screening became widely available, and the absolute difference in median PSA levels associated with this variant is quite small (0.2-0.3 ng/mL^29^) and thus likely clinically insignificant.

Previous investigators of the HPFS cohort have reported on the increased risk of prostate cancer among men with either a father or brother with prostate cancer (relative risk≈1.8) and that a family history of breast cancer is independently associated with lethal prostate cancer.^11,42^ In addition, they showed that men whose relatives were diagnosed at a younger age (<60 years) had a higher risk of prostate cancer than those whose relatives were diagnosed at an older age (≥60 years). We defined early-onset prostate cancer similarly to previous studies^13,41,42^; however, in our study we did not find an association between a family history of early-onset disease and lethal prostate cancer. This may be explained by the relatively small number of participants with a family history of early-onset disease (n = 78) and differences between familial risk of nonlethal vs lethal prostate cancer. Most recently, the combination of family history and PRS has been shown to stratify lifetime risk of developing prostate cancer among the overall HPFS cohort.^48^

Our study has several limitations. Family history data were self-reported by study participants, and family history was only available for first-degree relatives. However, self-reported data have been shown to be highly accurate (92% in a Swedish population^49^; 86% in a US population^50^). While reporting of cancer family history has been shown to be less accurate and variable by cancer type,^51-53^ the accuracy of self-reported data among this cohort composed of health professionals is expected to be high. Family history was assessed infrequently and has not been reported since 2008 in the HPFS cohort, and thus it is possible that family history is underreported and that the true association between family history and prostate cancer‒specific death is not adequately assessed. The statistical approach of including family history as a time-varying covariate aims to address this issue, but underreporting is difficult to control methodologically. The primary end point of prostate cancer‒specific death was obtained from multiple objective sources. A study population exclusively composed of health professionals can, however, also introduce selection bias because they could have better access to health care and early detection. The limited number of participants with the primary end point (n = 55) constrains the generalizability of the estimates derived from the models presented in this study and precluded testing for interactions between family history and the PRS or risk SNPs. Our study was underpowered to demonstrate an independent association for each component of the family history separately, despite the combined family history measure yielding the best model fitness. The study is also limited by the absence of germline genetic testing for rare pathogenic mutations (eg, *BRCA1/2*), which are expected to affect a small number of patients but would potentially enhance risk assessment. The diagnostic biopsy data available lacked the granularity to incorporate the percentage of biopsy cores positive or core percentage tumor involvement into our analysis, and thus it is possible that a portion of the participants designated as favorable intermediate risk in our analysis may have had NCCN unfavorable intermediate-risk disease by the > 50% cores positive criterion. To account for this, we performed a subgroup analysis on the participants with NCCN low-risk prostate cancer who could be confidently classified as AS eligible under stricter criteria, and this demonstrated an even stronger association between the heritable risk factors and lethal prostate cancer. Another limitation is the predominantly White study population (98%). This limits the generalizability of our findings to other populations such as Black/African Americans whose rate of prostate cancer‒specific death is more than twice as high compared to Whites^54^; further investigation to validate our findings in diverse populations is needed. Finally, we acknowledge that the cohort represents a population of largely treated patients (with only approximately 10% of patients initially managed by watchful waiting or AS), and thus, validation in contemporary AS cohorts is warranted.

## CONCLUSIONS

We found that participants with favorable-risk prostate cancer with a family history of prostate, breast, or pancreatic cancer and/or a 19q13 germline risk allele have an elevated risk of prostate cancer‒specific death. Further research is warranted to validate these findings in additional, diverse study populations. If validated, these findings have implications for how family and germline genetic risk SNPs should be factored into clinical decision-making around favorable-risk prostate cancer.
